# Retraction Note: α-santalol inhibits the angiogenesis and growth of human prostate tumor growth by targeting vascular endothelial growth factor receptor 2-mediated AKT/mTOR/P70S6K signaling pathway

**DOI:** 10.1186/s12943-022-01700-y

**Published:** 2022-12-15

**Authors:** Sarita Saraswati, Shakti Kumar, Abdulqader A. Alhaider

**Affiliations:** 1 Camel Biomedical Research Unit, College of Pharmacy and Medicine, King Saud University, Riyadh, Kingdom of Saudi Arabia; 2 Bioinformatic Centre, North-Eastern Hill University, Shillong, 793022 India; 3 Department of Physiology, College of Medicine, King Saud University, Riyadh, Saudi Arabia

**Retraction Note: Mol Cancer 12, 147 (2013)**

**https://doi.org/10.1186/1476-4598-12-147**

The Editor in Chief has retracted this article because of significant concerns regarding a number of Figures presented in this work, which question the integrity of the data. There were found to be overlapping images in figure 3A between the Vandetanib and the Sunitinib and in figure 7A between the PC-3 α-santalol 10μM and the PC-3 α-santalol 20μM. Images in Figure 3C were found to overlap with images found in Figure 5 of a previously published article [1]. Images in Figure 5A were found to overlap with images found in Figure 4A of a previously published article [2]. Images in Figure 5E were found to overlap with images found in Figure 3D of a previously published article [3].

Sarita Saraswati has not responded to any correspondence from the editor/publisher about this retraction. The Publisher has not been able to obtain a current email address for authors Shakti Kumar and Abdulqader A Alhaider.
